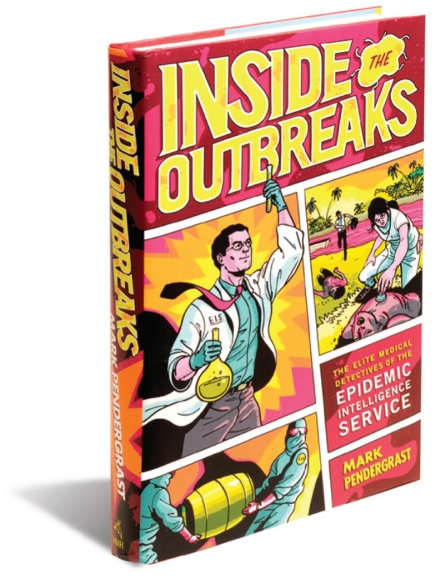# Inside the Outbreaks: The Elite Medical Detectives of the Epidemic Intelligence Service

**Published:** 2011-01

**Authors:** Beth E. Meyerson

**Affiliations:** *Beth Meyerson is the President/CEO of Policy Resource Group, LLC in Indianapolis Indiana. She has a faculty appointment at Walden University in the School of Public Policy & Administration, College of Social and Behavioral Sciences. Meyerson is co-author of the book* Ready to Go: The History and Contributions of U.S. Public Health Advisors *(ASHA, 2008)*

The story begins with a classic public health parable: Two physicians observe people floating down the Brown River. Some were alive and struggling, but with eyes glazed. The doctors spring into action, pulling as many people as possible from the river. As the flow of bodies continues unabated, the epidemiologist jumps out of the water and begins to run upstream. Her colleague protests, “For God’s sake, help me save these people!” Instead, she ventures upstream to determine the cause of this carnage. With this parable, every reader gains a basic appreciation for the important yet ethically challenging work of public health: Despite the significance of immediate need for rescue and treatment, the cause must ultimately be understood so as to prevent further devastation.

*Inside the Outbreaks: The Elite Medical Detectives of the Epidemic Intelligence Service* shares the stories of public health investigations conducted by the Epidemic Intelligence Service (EIS) officers of the Centers for Disease Control and Prevention since their advent in 1951. The history is told through a series of investigations presented in a rapid succession, bespeaking the range and pace of public health challenges facing this elite scientific corps of men and women.

The EIS was established as a training program for physicians and later expanded to include a variety of professions linked with public health: nurses, veterinarians, dentists, statisticians, laboratorians, epidemiologists, social scientists, and attorneys. This scientific resource was developed and nationally executed in response to the paucity of public health capacity in state and local health departments. As with the Public Health Advisors who came before them, the EIS cadre has been a lithe, public health–trained workforce that can be detailed for service throughout the world. Both programs are critical to our global community health, and when their forces are combined, their efforts result in amazing outcomes such as the eradication of smallpox.

*Inside the Outbreaks* presents a dizzying array of stories from a cross section of EIS officers. The reader is introduced to the history of this unique troupe of scientists who have been indelibly and culturally imprinted by founder Alexander Langmuir. The book is divided into three sections (inception of the program, the “golden age,” challenges of the present) to provide some sense of the program’s development and challenge. As a history, it is more valuable as one that tells disease stories than one that tells the history of the EIS. There is a cursory nod to the evolution of EIS composition in terms of participating professions as well as officer diversity by sex, race/ethnicity, and nationality of membership. The reader does quickly glimpse, however, the various program iterations and can appreciate the enduring esprit de corps among EIS officers. Interviews with a selection of current and former EIS officers inform the work, as did document review and the author’s field experience in Niger that was inserted at the end of the volume.

The value of this story cannot be overstated. We are reminded not only of the importance of key public health policies, such as vaccination and disease reporting, but also of the role of EIS officers in the discovery of diseases themselves: their etiology, transmission, and treatment. The reader learns about lifesaving inventions emerging from field investigations, such as the Safe Water System—a simple yet genius invention that prevents recontamination of water by hands. Further, this book describes the challenging conditions of EIS work, the personal risk undertaken while investigating dangerous conditions, and the flexibility and resolve needed by EIS officers to reach remote populations. Those who are looking for exciting examples of public health in action can find them here.

An important policy story is also told about doing the work of public health in the political context, with the inevitable invisibility of this work and, by extension, the EIS program. We learn the litany of investigations—yet it is less important who conducts them than it is that they are in fact conducted. One can easily see how the field scientists who have been sleuthing and solving our most intractable public health puzzles can be forgotten. *Inside the Outbreaks* soberly reminds us that regulatory outcomes do not necessarily follow public health discoveries or even the agendas that emerge from such discoveries.

*Inside the Outbreaks* is a good read for the public health audience, those engaged in health policy, and the general public. Mark Pendergrast’s writing style allows access to a seemingly unending series of complex health issues. The reader becomes patently aware of the history and importance of key public health policies such as vaccination and disease reporting. The uninitiated will never again look at food, water, vaccinations, sex, and bugs in the same way.

## Figures and Tables

**Figure:**